# MicroRNA-378a-3p prevents initiation and growth of colorectal cancer by fine tuning polyamine synthesis

**DOI:** 10.1186/s13578-022-00930-3

**Published:** 2022-12-01

**Authors:** Ningning Liu, Tianpeng Zhang, Clifford J. Steer, Guisheng Song

**Affiliations:** 1 Department of Medicine, University of Minnesota, Minneapolis, MN 55455 USA; 2 Department of Genetics, Cell Biology and Development, University of Minnesota, Minneapolis, MN 55455 USA; 3 Division of Gastroenterology, Hepatology and Nutrition, University of Minnesota, 516 Delaware Street SE, Minneapolis, MN 55455 USA

**Keywords:** Ornithine decarboxylase inhibitor, c-Myc signaling, Non-coding RNA

## Abstract

**Background:**

Inhibitors of ornithine decarboxylase (ODC) are effective at preventing colorectal cancer (CRC). However, their high toxicity limits their clinical application. This study was aimed to explore the potential of microRNAs (miRNAs) as an inhibitor of ODC.

**Methods:**

miRNA array was used to identify dysregulated miRNAs in CRC tumors of mice and patients. Azoxymethane (AOM)/Dextran Sodium Sulfate (DSS) were used to induce CRC in mice. miRNA function in carcinogenesis was determined by soft-agar colony formation, flow cytometry, and wound healing of CRC cells. Mini-circle was used to deliver miRNA into colons.

**Results:**

MiRNA profiling identified miR-378a-3p (miR-378a) as the most reduced miRNA in CRC tumors of patients and mice treated with AOM/DSS. Pathway array analysis revealed that miR-378a impaired c-MYC and ODC1 pathways. Further studies identified *FOXQ1* (forkhead box Q1) and *ODC1* as two direct targets of miR-378a. FOXQ1 activated transcription of *c-MYC,* a transcription activator of *ODC1.* In addition to directly targeting *ODC1*, miR-378a also inhibited expression of *ODC1* via the FOXQ1-cMYC axis, thereby inhibiting polyamine synthesis in human CRC cells. Phenotypically, by reducing polyamine synthesis, miR-378a induced apoptosis and inhibited proliferation and migration of CRC cells, while disrupting the association of miR-378a with *FOXQ1* and *ODC1* offset the effects of miR-378a, suggesting that *FOXQ1* and *ODC1* were required for miR-378a to inhibit CRC cell growth. MiR-378a treatment robustly prevented growth of HCC by inhibiting polyamine synthesis in AOM/DSS mice.

**Conclusion:**

MiR-378a prevents CRC by inhibiting polyamine synthesis, suggesting its use as a novel ODC inhibitor against CRC.

**Supplementary Information:**

The online version contains supplementary material available at 10.1186/s13578-022-00930-3.

## Background

Colorectal cancer (CRC) is the second leading cause of cancer-related death worldwide [1, 2]. Despite the improvement of current treatment strategies including surgical resection, radiotherapy and chemotherapy, 5-year overall survival rate of patients with CRC in U.S is roughly 65% because of tumor recurrence and/or metastasis [2, 3]. Therefore, it is imperative to perform a comprehensive understanding of molecular variables that control pathways involved in colon carcinogenesis and identify new and effective therapeutic agents with minimal adverse effects and toxicity to other organs.

Many molecular drivers of CRC have been reported over the years and some therapeutic agents based on these drivers have entered into clinical trials [4]. ODC1, the rate-limiting enzyme in polyamine synthesis, is another new and important therapeutic target for the treatment of CRC [5]. Polyamines are a class of small polycationic metabolites that are involved in cell growth and proliferation [6]. Although ODC1 activity is required for normal growth, it is elevated in many types of human cancers such as CRC and liver cancer [7]. In cancer, polyamine synthesis is increased primarily due to activation of polyamine biosynthetic enzymes [6, 8], which drives malignant transformation of cells and tumor progression. Activation of polyamine biosynthesis is controlled by multiple oncogenic pathways including RAS and c-MYC [9]. These oncogenic events frequently occur in CRC [10]. Polyamine synthesis is heavily regulated by c-MYC. c-MYC is able to activate expression of genes controlling polyamine synthesis including *ODC1*. [11] Inhibitors of polyamine synthesis and polyamine transport have been tested in preclinical and clinical studies [6, 8]. Despite some promising observations during the clinical trial, polyamine inhibitors have generally been proven to be disappointing [11, 14], except for difluoromethylornithine (DFMO) that is effective at preventing recurrent CRC [12, 13]. However, DFMO has severe side effects including hearing loss [14], which limits its clinical application.

MicroRNAs (miRNAs) are a class of small non-coding RNAs that function by binding to the 3'-untranslated regions (3'-UTR) of specific mRNAs, which leads to mRNA cleavage or translational repression [15, 16]. miRNAs fine-tune gene expression in mammalian cells to maintain physiological homeostasis. This exquisite ability of miRNAs makes them an ideal candidate regulator to precisely modulate level of polyamines to prevent initiation of CRC but still meet the needs for growth of normal cells. By comparing miRNA profile of CRC patients and normal individuals, we identified miR-378a as the most downregulated in both human and mouse CRC. Further studies revealed that miR-378a was an inhibitor of polyamine synthesis by modulating the FOXQ1-cMYC-ODC1 axis. In this study, we tested the hypothesis that miR-378a prevents CRC development by repressing overproduction of polyamine.

## Results

### MiR-378a is the most reduced miRNA in CRC tumors of patients and AOM/DSS mice.

**Fig. 1:**
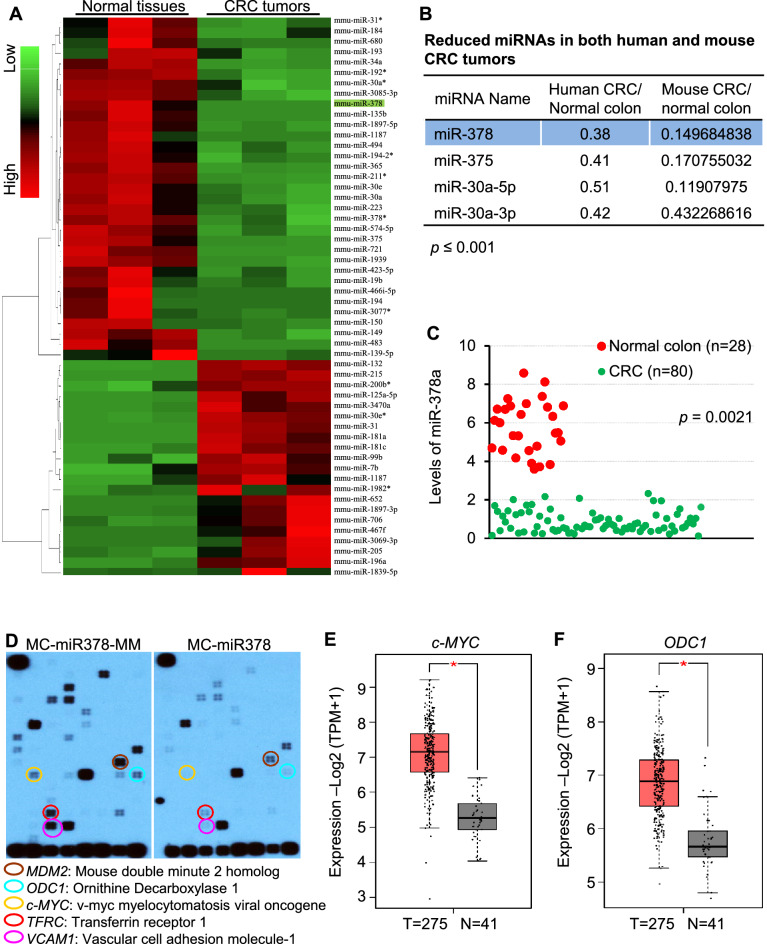
miR-378a is reduced in CRC tumors of patients and AOM/DSS mice. **A** 54 differentially expressed miRNAs in CRC tumors of AOM/DSS mice (*n* = 3). Normal colons from wild-type mice (*n* = 3) served as the control. Fold change ≥ 2 and *p* ≤ 0.05. **B** Four miRNAs that are reduced in CRC tumors of patients and AOM/DSS mice. **C** qRT-PCR confirming that miR-378a was significantly reduced in 80 CRC tumors versus 28 normal colon tissues. **D** Reduced expression of *Mdm2*, *Odc1*, *c-Myc*, *Tfrc*, and *Vcam1* in mouse colorectal cancer CT26.WT cells. Oligo Signal Transduction PathwayFinder MicroArray (Qiagen) was used to identify signaling pathways altered by miR-378a. **E**, **F** Reduced mRNA levels of *c-MYC* and *ODC1* in 275 CRC tumors versus 41 normal colon tissues in TCGA database. TPM: transcripts per million. Expression levels were shown as Log2 (TPM + 1). *P*-value was determined by DEseq. Data represent mean ± SEM. *p < 0.05 (**A**–**C**: two-tailed student’s *t* test)

CRCs arise from one or a combination of three different mechanisms, namely chromosomal instability (CIN), CpG island methylator phenotype (CIMP), and/or microsatellite instability (MSI) [4]. Most of the miRome studies on colon cancer focus on the colon tumor and adjacent non-tumor tissue [17–19]. Such a strategy only identified differentially expressed miRNAs triggered by CIN, CIMP or MSI since cells from both cancerous colon tissues and adjacent normal tissues have the same genome DNA. Importantly, the procedure of tumor resection is a potentially strong contributor to the variability of data among different studies, indicating that it is important to compare miRNAs between CRC patients and normal individuals. Previously, we have identified 37 differentially-expressed miRNAs in 80 colon tumors compared to 28 normal mucosa samples [20]. We further analyzed miRNA profiles from normal colon tissues and CRC tumors from AOM/DSS mice and identified 54 differentially-expressed miRNA (Fig. 1A) [21]. To identify miRNAs that are altered in CRC tumors of both patients and mice, we correlated altered 54 miRNAs in AOM/DSS mice with 37 miRNAs that are differentially-expressed in CRC patients [20]. This correlation analysis identified four miRNAs that were downregulated in both human and mouse CRC tumors (Fig. 1B). miR-378a-3p (miR-378a) was identified as the most reduced among four under-expressed miRNAs in CRC tumor, leading us to focus on miR-378a. qRT-PCR analysis of 28 normal colon tissues and 80 colon tumors further validated that miR-378a expression was robustly reduced in colon tumors versus normal colon tissues (Fig. 1C).

To determine the role of miR-378a in regulating CRC, we overexpressed miR-378a in mouse colorectal adenocarcinoma CT26.WT cells and determined expression of 117 genes related to the onset, development, and progression of tumorigenesis using the Signal Transduction PathwayFinder MicroArray (Qiagen). For this purpose, we constructed an expression vector of miR-378a by cloning miR-378a precursor into mini-circle vectors (MC). MCs are episomal DNA vectors that are produced as circular expression cassettes devoid of any bacterial plasmid DNA backbone [22]. Their small molecular size enables more efficient delivery and offers sustained expression over several weeks compared to standard plasmid vectors that only express for a few days after injection into mice [22]. This vector was referred to as MC-miR378. To rule out a non-specific effect of the plasmid, we generated a miR-378a mis-matched-expression vector by mutating the seed region of miR-378a (MC-miR378-MM). Transfection of MC-miR378 into CT26.WT cells significantly inhibited expression of five genes including *Mdm2* (mouse double minute 2 homolog), *Odc1*, *c-Myc*, *Tfrc* (transferrin receptor 1) and *Vcam1* (vascular cell adhesion molecule-1) (Fig. 1D). Among five genes reduced by miR-378a, no significant change in levels of *MDM2*, *TFRC*, and *VCAM1* was observed in human CRC tumors (*n* = 275) versus normal colon specimen (*n* = 41) from TCGA database (Additional file 1: Fig. S1A–C), while levels of *c-MYC* and *ODC1* were significantly increased (Fig. 1E, F). In summary, level of miR-378a is inversely correlated with mRNA levels of *ODC1* and *c-MYC* in both human and mouse CRC tumors.

### miR-378a inhibits expression of *ODC1* by binding to its 3’UTR

**Fig. 2:**
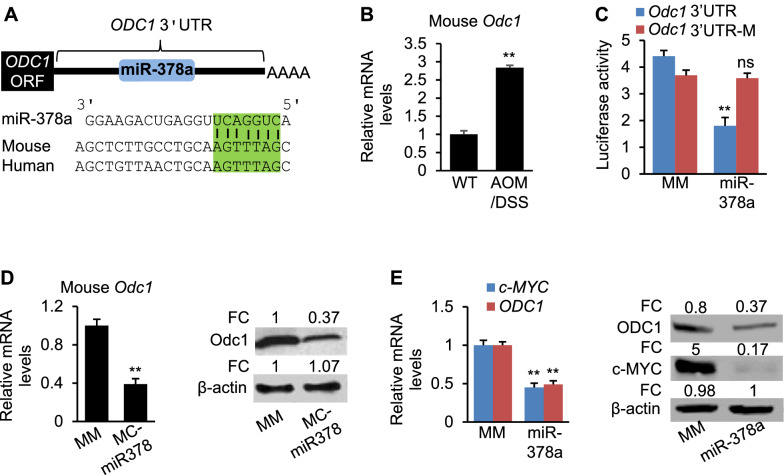
miR-378a inhibits expression of *Odc1* via binding to its 3’UTR. **A** Graphic representation of the conserved miR-378a binding motif within the 3’UTRs of *ODC1* between human and mouse. **B** mRNA levels of *Odc1* in CRC tumors of AOM/DSS mice (*n* = 6) and normal colon tissues of WT mice (*n* = 6). **C** miR-378 significantly reduced luciferase activity of the luciferase reporter construct containing the 3’UTR of *Odc1* with either wild-type or mutated miR-378a binding site. Mutation of the miR-378 binding site within the 3’UTR of *Odc1* canceled the ability of miR-378a to inhibit luciferase activity. **D** mRNA and protein levels of *Odc1* in AOM/DSS mice injected with MC-miR378 or MC-miR378-MM (MM, control). FC: fold change. **E** mRNA and protein levels of *ODC1* and *c-MYC* in DLD-1 cells transfected with MC-miR378 or MC-miR378-MM. FC: fold change. Data represent mean ± SEM. **p < 0.01, ns: no significance (**B**–**E**: two-tailed student’s *t* test)

It is known that dysregulated activities of ODC1 and c-MYC are important promoters of CRC. We, therefore, hypothesized that miR-378a was a potential tumor suppressor by repressing *ODC1* and *c-MYC* expression. We next determined the molecular mechanism by which miR-378a inhibits expression of *ODC1* and *c-MYC*. Combining bioinformatic prediction and mining of Ago HITS-CLIP database (high-throughput sequencing of RNAs isolated by crosslinking immunoprecipitation from argonaute protein complex) [23], we identified a conserved binding site of miR-378a within the 3’UTR of *ODC1* (Fig. 2A). Consistent with reduced miR-378a, expression of *Odc1* was significantly increased in CRC tumors of AOM/DSS mice (Fig. 2B). To determine if miR-378a inhibits *Odc1* directly by binding to the 3’UTR of *Odc1*, a luciferase reporter was employed, in which the 3’UTR containing wild-type or mutated miR-378a binding site was embedded into the downstream of the luciferase. Indeed, the luciferase activity was reduced by miR-378a in HCT116 cells (Fig. 2C), whereas mutation of the miR-378a binding site within the 3’UTR of *Odc1* impaired the ability of miR-378a to reduce luciferase activity (Fig. 2C), suggesting the direct repression of miR-378a on *Odc1.* Injection of miR-378a into mice also repressed mRNA and protein levels of *Odc1* (Fig. 2D). In human DLD-1 cells, overexpression of miR-378a led to reduced mRNA and protein levels of *c-MYC* and *ODC1* (Fig. 2E). In summary, miR-378a is able to inhibit expression of both murine and human *ODC1* by binding to their 3’UTRs*.*

### MiR-378a indirectly inhibits expression of *c-MYC* by inhibiting *FOXQ1*.

**Fig. 3:**
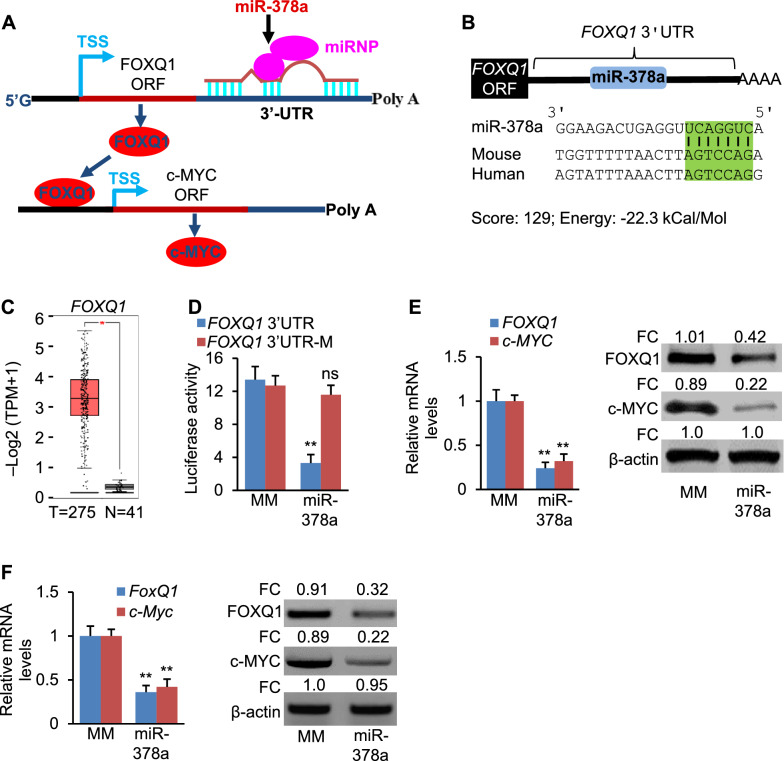
miR-378a inhibits *c-MYC* transcription by directly targeting *FOXQ1*. **A** A proposed mechanism by which miR-378a inhibits transcription of *c-MYC* by directly targeting *FOXQ1* that serves as a transcription activator of *c-MYC*. **B** The predicted miR-378a binding motif within the 3’UTRs of human and mouse *FOXQ1*. **C** mRNA levels of *FOXQ1* in CRC tumors of 275 patients versus 41 normal colon tissues in TCGA database. TPM: transcripts per million. Expression levels were shown as Log2 (TPM + 1). *P*-value was determined by DEseq. **D** Luciferase activities of the reporter constructs containing the 3’UTR of *FoxQ1* with either WT or mutated miR-378a binding site after treatment with MC-miR378. HCT116 cells transfected with the luciferase reporter vector and MC-miR378-MM served as a control. **E** mRNA and protein levels of *FOXQ1* and *c-MYC* in DLD-1 cells transfected with MC-miR378 or MC-miR-378-MM (control). FC: fold change. **F** Protein and mRNA levels of *FoxQ1* and *c-Myc* in CT26.WT cells transfected with MC-miR378 or MC-miR-378-MM (control). FC: fold change. Data represent mean ± SEM. **p < 0.01, and ns: no significance (**D**–**F** two-tailed student’s *t* test)

Although miR-378a significantly inhibited expression of *c-MYC* (Fig. 1D), no binding motif for miR-378a was identified within the 3'UTR of *c-MYC*. Luciferase assay confirmed that miR-378a had no binding site within the 3’UTR of *c-MYC* (Additional file 1: Fig. S2A, B). We, therefore, speculated that miR-378a inhibited transcription of *c-MYC* expression via directly targeting a transcription activator that has a binding site within the promoter of *c-MYC*. To test this speculation, we used MatInspector software to scan the promoter of *c-MYC* and identified a highly conserved binding motif for FOXQ1 (Fig. 3A) [24]. FOXQ1 is an oncogenic transcription factor that binds to GTTT core motif [25]. Furthermore, combining bioinformatic prediction and mining of Ago HITS-CLIP database, we identified a potential binding site of miR-378a within the 3’UTR of *FOXQ1* (Fig. 3B). Level of *FOXQ1* was increased in CRC patients (Fig. 3C)*.* These findings led us to speculate that miR-378a inhibited expression of *c-MYC* by repressing transcription of *FOXQ1* (Fig. 3A). miR-378a significantly reduced luciferase activity of the construct containing the 3’UTR of *FOXQ1*, whereas disrupting the interaction between miR-378a and *FOXQ1* by mutating miR-378a binding site prevented miR-378a from repressing luciferase activity (Fig. 3D). Overexpression of miR-378a in DLD-1 cells reduced mRNA and protein levels of *FOXQ1* and *c-MYC* (Fig. 3E). In murine CT26.WT cells, overexpression of miR-378a significantly inhibited expression of *FoxQ1* and *c-Myc* (Fig. 3F). Together, *FOXQ1* is a direct target of miR-378a.

**Fig. 4:**
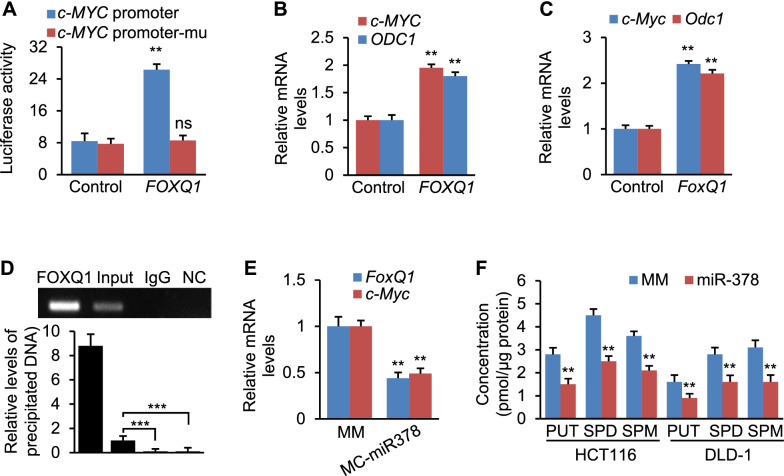
FOXQ1 activates transcription of *c-MYC*. **A** Luciferase activity of the reporter construct containing *c-MYC* promoter with either WT or mutated FOXQ1 binding site after treatment with *FOXQ1* expression vector or empty vector (control). **B** mRNA levels of *c-MYC* and *ODC1* in DLD-1 cells transfected with *FOXQ1* expression vector or empty vector (control). **C** mRNA levels of *c-Myc* and *Odc1* in CT26.WT cells transfected with *FoxQ1* expression vector or empty vector (control). **D** ChIP assay was performed using genomic DNA isolated from DLD-1 cells; and the binding of FOXQ1 to the endogenous promoter of *c-MYC* was detected using an FOXQ1 antibody; N.S.: non-specific control, which is located 10 kb downstream of the predicted FOXQ1 binding site. **E** mRNA levels of *c-Myc* and *Odc1* in AOM/DSS mice injected with MC-miR378 or MC-miR378-MM (MM, control). **F** Levels of putrescine (PUT), spermidine (SPD) and spermine (SPM) in DLD-1 and HCT116 cells after transfection of MC-miR378 or MC-miR378-MM (control). Data represent mean ± SEM. **p < 0.01, ***p < 0.001, and ns: no significance (**A**–**C**, **E**, **F**: two-tailed student’s *t* test; **D**: two-way ANOVA test)

We next determined if FOXQ1 was a transcription activator of *c-MYC*. Luciferase assay was used to evaluate if FOXQ1 was able to inhibit transcription of *c-MYC*. Overexpression of *FOXQ1* significantly increased activity of the *c-MYC* promoter (Fig. 4A). To determine if the binding site for FOXQ1 within the *c-MYC* promoter was required for increased expression of *c-MYC*, we mutated the FOXQ1 binding site within the *c-MYC* promoter. As expected, this mutation prevented FOXQ1 from driving transcription of *c-MYC* (Fig. 4A). Overexpression of *FOXQ1* strongly enhanced transcription of both *c-MYC* and *ODC1* in DLD-1 cells (Fig. 4B). Overexpression of *FoxQ1* in mouse colon cells lead to increased *c-Myc* and *Odc1* (Fig. 4C). As revealed by chromatin immunoprecipitation (ChIP), DNA fragments containing FOXQ1 binding motif within the *c-MYC* promoter were immune-precipitated from genomic DNA from DLD-1 cells by an FOXQ1 antibody (Fig. 4D), suggesting that FOXQ1 was able to physically bind to the promoter of *c-MYC*. Delivery of miR-378a into mice reduced mRNA levels of *FoxQ1* and *c-Myc* (Fig. 4E). Our hypothesis is that miR-378a inhibits polyamine synthesis by directly targeting *ODC1* and blocking the FOXQ1-MYC-ODC1 axis. We next measured the effect of miR-378a on levels of polyamine in DLD-1 and HCT116 cells. As expected, miR-378a significantly reduced spermine (SPM), putrescine (PUT), and spermidine (SPD) in DLD-1 and HCT116 cells (Fig. 4F). In summary, FOXQ1 is a transcription activator of c-*MYC*. miR-378a inhibited polyamine synthesis by directly repressing *ODC1* and blocking the FOXQ1-c-MYC-ODC1 signaling.

### MiR-378a inhibited growth and proliferation of CRC cells via inhibiting polyamine synthesis.

**Fig. 5:**
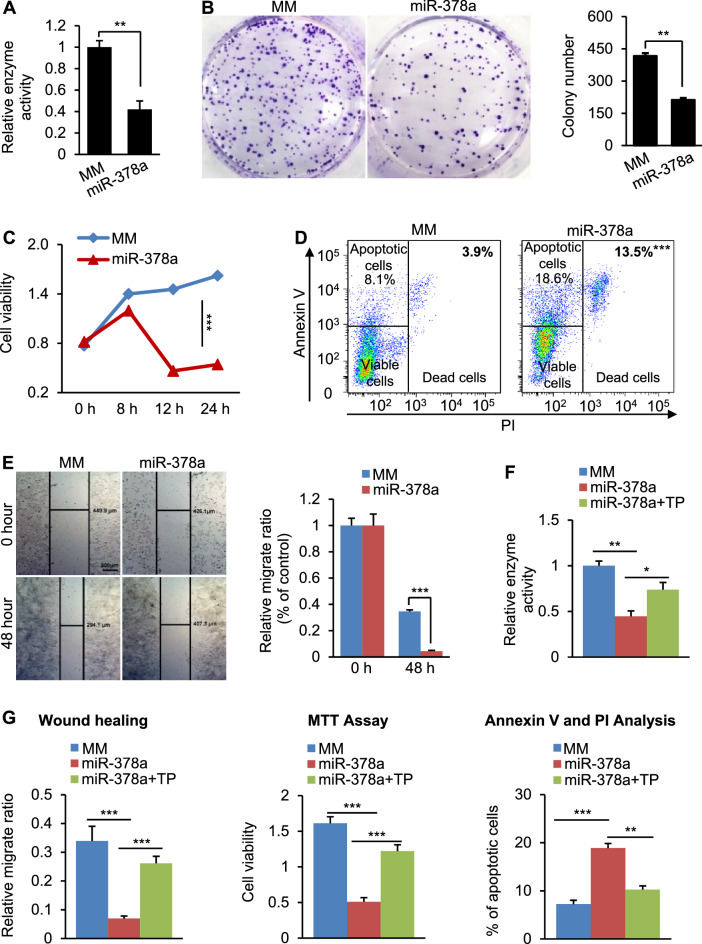
*ODC1* mediates the role of miR-378a in inhibiting proliferation and growth and inducing apoptosis of CRC cells. **A** Enzyme activity of ODC in DLD-1 cells transfected with MC-miR378 or MC-miR378-MM (control). **B** Soft agar colony formation assay revealed that miR-378a overexpression inhibited growth and viability of DLD-1 cells. DLD-1 cells were transfected with MC-miR378 or MC-miR378-MM (control). **C** Reduced viability of DLD-1 cells after MC-miR378 transfection, as revealed by MTT assay. DLD-1 cells treated with MC-miR378-MM served as the control. **D** miR-378a induced apoptosis of DLD-1 cells, as revealed by flow cytometry. A statistical graph of Annexin V-FITC/PI staining is shown. **E** Wound healing showing that miR-378a significantly impaired growth and migration of DLD-1 cells. **F** miR-378a treatment reduced ODC enzyme activity, while additional treatment of TPs of *ODC1* and *FOXQ1* impaired the ability of miR-378a to inhibit ODC activity. Three groups of DLD-1 cells were transfected with MC-miR378-MM (control), MC-miR378, or a combination of MC-miR378 and TPs of *ODC1* and *FOXQ1*. **G** miR-378a reduced proliferation and growth of DLD1 cells and induced apoptosis of DLD1 cells, while additional treatment of TPs of *FOXQ1* and *ODC1* counteracted the ability of miR-378a to repress proliferation and growth and induce apoptosis of DLD-1 cells, as revealed by Wound Healing, MTT assay, and flow cytometry analysis, respectively. Data represent mean ± SEM. *p < 0.05, **p < 0.01, ***p < 0.001 (**A**–**E**: two-tailed student’s *t* test,** F**–**G**: two-way ANOVA test)

To determine the role of miR-378a in tumorigenesis of CRC, we overexpressed miR-378a in DLD-1 cells, which led to reduced mRNA levels of *FOXQ1* and *ODC1* (Additional file 1: Fig. S3A). Overexpression of miR-378a significantly reduced ODC activity in DLD-1 cells and polyamine synthesis (Fig. 5A, Additional file 1: Fig. S3B), which subsequently inhibited their growth and viability, as revealed by colony formation and MTT assay (Fig. 5B, C). In addition to inhibiting proliferation and migration, miR-378a also significantly induced apoptosis and death of DLD-1 cells (Fig. 5D). Would healing assay further validated the inhibitory effect of miR-378a on growth of DLD-1 cells (Fig. 5E).

MiR-378a targets many genes simultaneously. We next determined whether the FOXQ1-MYC-ODC1-mediated polyamine synthesis, at least in part, was responsible for miR-378a to inhibit proliferation and induce apoptosis. For this purpose, we used a novel technique Target Protector morpholinos (TP) to prevent the interaction of miR-378a with *FOXQ1* and *ODC1*. [26] The morpholinos were complimentary to the miR-378a binding sites present within the 3’UTRs of *FOXQ1* and *ODC1* mRNAs, thereby preventing miR-378a from binding to the 3’UTRs of *FOXQ1* and *ODC1*. Specifically, three groups of DLD-1 cells were transfected with MC-miR378-MM, MC-miR-378 or a combination of MC-miR-378 and TPs of *ODC1* and *FOXQ1*. Overexpression of miR-378a reduced expression of *FOXQ1* and *ODC1*, while additional treatment of TPs of *FOXQ1* and *ODC1* impaired the ability of miR-378a to prevent their expression (Additional file 1: Fig. S3C). MiR-378a treatment reduced ODC activity and polyamine synthesis, while additional treatment of *FOXQ1* and *ODC1* TPs offset the effects of miR-378a (Fig. 5F, Additional file 1: Fig. S3D). Treatment of *FOXQ1* and *ODC1* TPs, which disrupted the interaction between miR-378a and 3’UTRs of *FOXQ1* and *ODC1*, impaired the ability of miR-378a to inhibit proliferation and migration and induce apoptosis of DLD-1 cells (Fig. 5G). In sum, the FOXQ1-MYC-ODC1 axis mediates the roles of miR-378a in inhibiting polyamine synthesis, preventing proliferation and growth, and inducing apoptosis of CRC cells.

### MiR-378a prevented overproduction of polyamine and growth of CRC in AOM/DSS mice

**Fig. 6:**
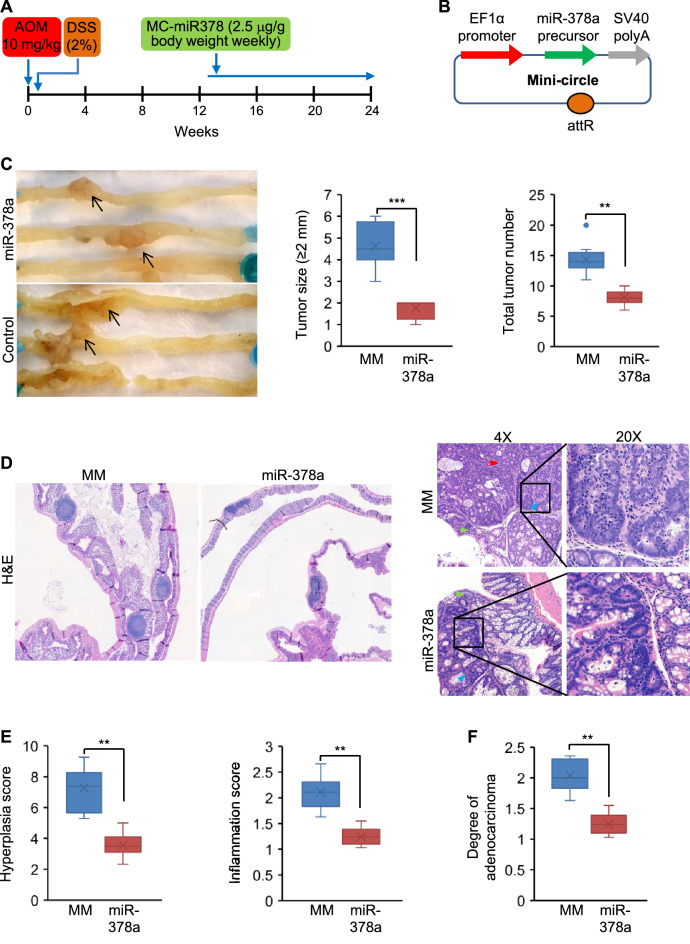
miR-378a significantly prevented CRC development in AOM/DSS mice. **A** Scheme for AOM/DSS-induced colon carcinogenesis in mice. Mice were kept on normal chow. Five-weeks old mice were given AOM, followed by 1% DSS in water for 4 days. At 13 weeks of age, mice were injected with MC-miR378-MM (*n* = 6, control) or MC-miR378 (*n* = 6) weekly for 10 weeks. **B** Diagram of miR-378a mini-circle expression vector (MC-miR378). **C** Representative photos of CRC tumors in mice treated with MC-miR378 and MC-miR378-MM. Black arrows indicate colon tumors. Both total tumor number and tumors with the size of ≥ 2 mm were reduced in AOM/DSS mice treated with MC-miR378. **D** H&E staining of colon from two groups of mice. Red arrowhead: acinar regions; green arrowhead: inflammation regions; and blue arrowhead: hyperplasia regions. **E**, **F** Scores of hyperplasia, inflammation and adenocarcinoma in AOM/DSS mice treated with MC-miR378 or MC-miR378-MM. Data represent mean ± SEM. **p < 0.01 and ***p < 0.001 (Fig. 6C, E–F: two-tailed student’s *t* test)

**Fig. 7:**
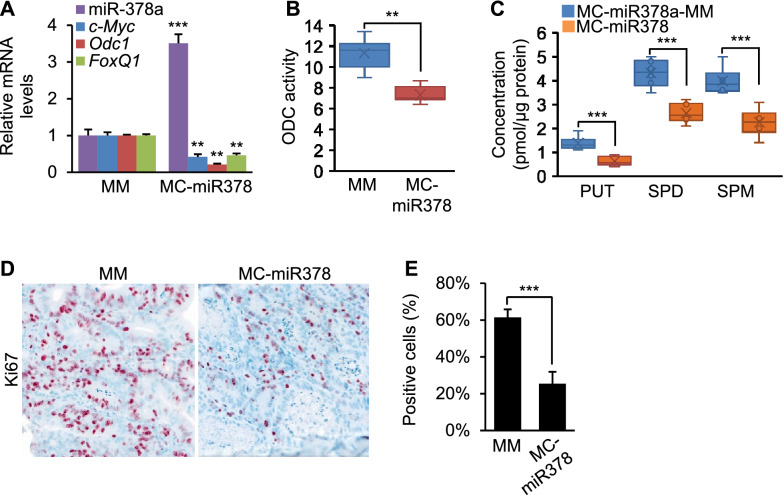
miR-378a reduced enzyme activity of ODC and proliferation of colon cells in AOM/DSS mice. **A** Levels of miR-378a and mRNA levels of *FoxQ1*, *c-Myc* and *Odc1* in AOM/DSS mice injected with MC-miR378 and MC-miR378-MM. **B** Reduced enzyme activity of ODC in AOM/DSS mice treated with MC-miR378. **C** Levels of putrescine (PUT), spermidine (SPD) and spermine (SPM) in AOM/DSS mice injected with MC-miR378 or MC-miR378-MM (control). **D**, **E** A significant reduction in proliferation of colon cells in AOM/DSS mice treated with MC-miR378, as revealed by Ki67 staining. Data represent mean ± SEM. **p < 0.01 and ***p < 0.001 (Fig. 7A-E: two-tailed student’s *t* test)

Carcinogen-induced colon cancer in mice simulates the phases of initiation and progression of tumor that occur in humans [27]. To evaluate the potential of miR-378a to inhibit CRC in vivo, we exposed mice to AOM and DSS to induce CRC (Fig. 6A). [27] The AOM/DSS mice recapitulate a chemical-induced carcinogenesis, inflammatory effects, colitis-driven initiation stage and sequential tumor progression [27]. Therefore, we next determined gain-of miR-378a function against CRC in AOM/DSS mice. The small molecular size of MCs enables efficient delivery and offers a sustained expression for a few weeks, as described above. We therefore used MC-miR378, as diagramed in Fig. 6B, to overexpress miR-378a in mice. Two groups of AOM/DSS mice were injected with either MC*-*miR378-MM (control) or MC*-*miR378 at a dose of 2.5 μg/g by tail vein weekly for ten weeks. Injection of MC-miR378 into AOM/DSS mice led to increased miR-378a in the colon (Additional file 1: Fig. S4). MiR-378a treatment resulted in a significant reduction in tumor size and number (Fig. 6C). We next performed the histopathological evaluation of intestinal inflammation, hyperplasia and tumorigenicity using H&E staining. Consistent with previous reports [28], diffuse mucosal hyperplasia was observed in the distal colon of AOM/DSS mice (control) (Fig. 6D). In contrast, no or much less hyperplasia was observed in the colonic mucosae of AOM/DSS mice treated with miR-378a (Fig. 6D). Inflammation is a major driver of CRC development in AOM/DSS mice. A significant reduction in inflammation and hyperplasia score was observed in miR-378a-treated AOM/DSS mice (Fig. 6E). MiR-378a treatment also reduced adenocarcinoma (Fig. 6F). These observations indicated that miR-378a was able to alleviate colonic hyperplasia and adenocarcinoma. H&E staining revealed that CRC tumors were well differentiated with an acinar pattern in MC-miR378-treated AOM/DSS mice, whereas CRC tumors in control AOM/DSS mice exhibited poor differentiation, indicating the strong inhibitory effect of miR-378a on colon cancer development (Fig. 6D). Mechanistically, miR-378a led to reduced mRNA levels of *FoxQ1*, *c-Myc* and *Odc1*, enzyme activity of ODC and polyamine synthesis (Fig. 7A-C). K67 staining (general marker of cell proliferation) confirmed the inhibitory effect of miR-378a on growth of colon cancer, which was reflected by a significant reduction in proliferating colon cells (Fig. 7D-E). In summary, miR-378a maintains an appropriate level of polyamine by precisely fine tuning polyamine synthesis, which subsequently prevents CRC development.

## Discussion

Both c-MYC signaling and polyamine synthesis are frequently activated in human cancers, especially during the early-stage development of CRC [29, 30]. Targeting oncogenic *c-MYC* and *ODC1* has emerged as a strategy for cancer treatment [31, 32]. However, their clinical applications have been impeded due to serious adverse effects and low effectiveness. Advance in CRC treatment requires the development of new, specific, and highly targeted therapies. Recent studies of miRNAs brought polyamine metabolism and c-MYC signaling back to the attention, providing a potential correlation between miRNAs and c-MYC-ODC1 axis in tumorigenesis. In this study, we uncover a significant reduction in miR-378a that inhibits c-MYC signaling and ODC1-mediated polyamine biosynthesis, highlighting the role of miR-378a as a potent inhibitor of abnormal polyamine synthesis and CRC.

This study provides several novel insights into the biology and therapeutic relevance of miR-378a for the treatment of CRC. First, we established that miR-378a was significantly down-regulated in CRC tumors of patients and mice, suggesting that our findings could be applied to patients. miRNAs are naturally-occurring noncoding RNAs that fine tune expression of their targets [33]. This characteristic of miRNAs led to our speculation that miR-378 was able to precisely maintain level of polyamine and represents a unique and novel therapeutic agent with less adverse effects compared to chemically synthesized ODC1 and c-MYC inhibitors.

Second, miR-378a served as a potent inhibitor of polyamine biosynthesis by directly targeting *ODC1* and blocking the FOXQ1-MYC-ODC1 axis. In addition to directly inhibiting *ODC1*, miR-378a also exerted its inhibitory effect on *ODC1* by blocking the FOXQ1-MYC axis, suggesting that miR-378a could modulate polyamine synthesis at multiple layers. An increase in enzymes controlling polyamine synthesis such as ODC1 leads to increased polyamines, which are thought to maintain the transformed phenotype and tumor development [9]. Intriguingly, extravagant polyamine itself could exert a major damaging effect on cell viability [34], suggesting that precisely maintaining polyamine level is required for normal growth of cells and prevention of transformation of normal cells. MiR-378a is such a fine tuner to maintain an appropriate level of polyamine. In CRC cells, polyamine level is much higher than in normal cells due to the upregulation of *ODC1* from oncogenic signaling [5, 6]. As such, overexpression of miR-378a prevents polyamine level from extending beyond the toxic threshold, and still maintain a relatively reasonable level of polyamine favorable to support normal growth of cells. Thus, a potent inhibitor of ODC1 could potentially lead to a polyamine level that is lower than what is needed for normal grow of cells, providing a potential explanation of serious adverse effects of chemically-synthesized ODC inhibitors. Taken together, our findings highlight an important role of miR-378a in preventing imbalance of polyamine in colon cells.

Third, our study revealed a novel function of FOXQ1 that promotes abnormal activation of c-MYC signaling and polyamine synthesis in CRC. FOXQ1 is one of the FOX gene family and contains the core DNA binding domain, whereas its flanking wings are important for its sequence specificity [35]. FOXQ1 activates or represses transcription [36]. It is well described regarding the role of FOXQ1 in regulating hair follicle differentiation [37]. Recently, overexpression of *FOXQ1* is observed in CRC [38]. However, the underlying mechanism by which FOXQ1 promotes CRC is poorly characterized. Our findings for the first time revealed that FOXQ1 activated c-MYC signaling and polyamine synthesis, thereby driving CRC development. Indeed, survival analysis revealed a significant correlation between high *FOXQ1* mRNA and poor survival in CRC (Additional file 1: Fig. S5). We have established that *c-MYC* and *ODC1* are downstream effectors of FOXQ1 during the development of CRC. Aberrant expression of *c-MYC* and *ODC1* was observed in CRC (Fig. 1). Thus, FOXQ1-mediated activation of c-MYC signaling and polyamine synthesis is an intrinsic molecular phenotype of colon cancer.

Finally, our results indicate that miR-378a, via simultaneously inhibiting *ODC1*, *FOXQ1* and *c-MYC* pathways, represents a new therapeutic strategy for the treatment of CRC. As revealed by the Cancer Genome Atlas, activation of MYC-dependent transcription has been observed in nearly all types of CRC [10]. c-MYC functions as a master transcription factor in various biological processes by regulating many target genes, and excessive activation of c-MYC is one of the most prevalent oncogenic events in human cancers [39, 40]. In addition of c*-MYC* amplification, FOXQ1 was identified as another activator of c-MYC signaling. We showed that *FoxQ1* was increased in AOM/DSS mice. Notably, overall level of *FOXQ1* in 275 CRC patients was 8 times higher than that in 41 normal individuals (Fig. 3C). Overexpression of *FOXQ1* drove proliferation and growth of CRC cells. All these findings established both c-MYC and FOXQ1 were ideal therapeutic targets for CRC. Intriguingly, we established that FOXQ1 was a transcription activator of *c-MYC*, while *FOXQ1* was a direct target of miR-378a, indicating that miR-378a, in general, is an inhibitor of FOXQ1-cMYC cascade that is activated in CRC. Taken together, miR-378a is a master regulator to maintain normal level of polyamine by preventing abnormal activation of c-MYC and FOXQ1 signaling. MiR-378a-based therapeutics represents a promising approach to tailor CRC therapy with minimal toxicity.

## Material and methods

### Human CRC samples

Collection of human CRC tumors and normal mucosa were described in our precious publication [20]. All CRC and non-cancerous tissue specimens were evaluated by an experienced pathologist.

### AOM/DSS CRC mouse model

Wild-type male Balb/c mice at five-weeks old (Jackson Laboratory, *n* = 6) kept on a normal chow diet (SD) (Open Source D12450B: 10% Kcal fat) were injected with 10 mg/kg body weight of AOM (Azoxymethane) via intraperitoneal injection, followed by 2% DSS (Dextran Sodium Sulfate) in drinking water for 4 days. At 23 weeks of age, all mice developed CRC as described [27]. Growth of colon tumor was monitored with a VEVO 770 ultrasound micro-imaging system designed for use with small animals. Colons were collected for further analysis.

### miRNA profiling of CRC tumors in AOM/DSS mice

miRNA profiling data of CRC tumors of AOM/DSS mice were downloaded from Gene Expression Omnibus (GSE44988) [21]. GeneSpring (Agilent Technologies Genomics) was used to analyze miRNA profiles of three normal colon samples (wild-type mice) and three CRC tumor samples (AOM/DSS mice). Differentially expressed miRNAs were defined by a log-scale ratio ≤ 1 between paired samples with a *p* < 0.05. These criteria identified 54 differentially expressed miRNAs in CRC tumors of AOM/DSS mice. To identify miRNAs that are differentially-expressed in CRC tumors of both AOM/DSS mice and CRC patients, we used Microsoft Access 2016 to compare 54 differentially-expressed miRNAs in AOM/DSS mice with 37 differentially-expressed miRNAs in CRC patients in our previous study [20]. This correlation analysis identified four miRNAs that are reduced in CRC tumors of both AOM/DSS mice and CRC patients (Fig. 1B).

### Pathway array analysis

To identify miR-378a-regulated signaling pathways, we used Oligo GEarray Signal Transduction PathwayFinder MicroArray (Qiagen) to profile genes regulated by miR-378a in murine CRC cell line CT26.WT. Briefly, CT26.WT cells were transfected with miR-378a mimic or scramble control. 48 h post transfection, total RNA was isolated from two groups of CT26.WT cells using RNeasy Mini Kit (Qiagen). Synthesis of complimentary DNA, biotin labelling of cRNA, and hybridization of Mouse Oligo GEArray Mouse Signal Transduction PathwayFinder Microarray (OMM-014) were performed as described previously [41].

### Preparation of mini-circle expression vectors for miR-378a

Construction of mini-circle expression vector of miR-378a and preparation of mini-circles were performed based on the protocol as described previously [42]. Briefly, the murine genomic region containing the precursor of miR-378a was cloned into mini-circle vectors (System Biosciences, Cat. MN511A-1), referred to as MC-miR378. To further ascertain that reduced growth of CRC in MC-miR378-injected mice is due to miR-378a overexpression rather than toxicity caused by miR-378a, we generated a control scramble that differs from miR-378a in four mismatched base pairs (MC-miR378-MM). Mini-circles were prepared based on the manufacturer’s instructions.

### MC-miR378 treatment of AOM/DSS mice

Two groups of AOM/DSS mice was injected with either MC-miR378 (*n* = 6) or MC-miR378-MM (control, *n* = 6) at a dose of 1.5 μg/g MC-miR378 or MC*-*miR378-MM complexed with in vivo-jetPEI (Polyplus Transfection, Strasbourg, France) weekly for 10 weeks via tail vein injection. After 10 weeks of MC-miR378 treatment, colons were collected for further analysis.

### Cell culture and transfection

Colorectal cancer cell lines CT26.WT, HCT116 and DLD-1 (ATCC, Manassas, VA) were cultured in DMEM containing 10% FBS. All cells were split prior to establishment of confluence and incubated in a humidified incubator with 5% CO_2_ at 37℃. Lipofectamine 3000 (Invitrogen) was used to cell transfection following manufacturer’s protocol.

### Colony formation assay in soft agar

HCT116 or DLD-1 cells was plated in triplicate at 10,000 cells/mL in 0.35% Seaplaque agarose gel (Lonza) using 24 well plates. After two weeks, colonies were stained with crystal violet, photographed and counted.

### Cell viability assay

HCT116 or DLD-1 cells was plated in 96-well plates at 1,000/well. 48 h post transfection, cell viability was determined via MTT (3-(4, 5-Dimethylthiazol-2-yl)-2, 5- Diphenyltetrazolium Bromide) and detected absorbance at OD 570 nm by BioTek Synergy HTX multi-mode reader. The experiment was performed in triplicate.

### Apoptosis assay

Annexin V and propidium iodide (PI) (BD Pharmingen, San Diego, CA) were used to assess and quantify apoptosis of DLD-1 and HCT116 cells based on the manufacturer's protocols. 48 h after transfection, DLD-1 or HCT116 cells were detached by accutase (Invitrogen) and washed with cold PBS (pH 7.4). After five minutes of centrifuge at 2000 × g, cell were re-suspended in 100 μL of binding buffer (10 mM HEPES/NaOH, pH 7.4; 140 mM NaCl; and 2.5 mM CaCl_2_). The cell suspension with 1 × 10^5^ cells (100 μL) was then incubated with 5 μL of Alexa Fluor® 488 Annexin V and 1 μL of 100 μg/mL PI at room temperature in the dark for 15 min. After incubation, add 400 μL of 1 × Annexin-binding buffers, mix gently and then analyze with LSR II H1160 flow cytometer and analyzed in CellQuest software (BD Biosciences, San Diego, CA).

### Wound healing assay

24 h post transfection of DLD-1 or HCT116 cells, 200 μL plastic filter tip was used to make vertical scratches (a ‘wound’ of approximately 400 μm in diameter) and the medium was exchanged with fresh DMEM containing 2% FBS. Migration of cells were evaluated under an inverted microscope at 0, 24 and 48 h.

### Activity assay of ODC

Enzymatic activity of ODC1 was measured using CB6/DSMI method (Cucurbit[6]uril Hydrate, CB6 and trans-4-[4-(Dimethylamino)styryl]-1-methylpyridinium iodide, DSMI based on the manufacturer’s instruction (Catalog Number: 10316-OD) to measure the ability of ODC to convert ornithine to putrescine. L-ornithine monohydrochloride (Sigma, Catalog # O2375) was used as the substrate.

### Statistical analysis

GraphPad Prism Software® was used to perform statistical analysis. Data derived from cell-line experiments were presented as mean ± SEM and the statistical significance was evaluated via a two-tailed Student *t* test. ANOVA (Analysis of variance) was used to measure statistical difference among multiple groups. All the experiments were repeated at least three times. *p* < 0.05 was considered to be statistically significant.

## Supplementary Information

**Additional file 1: Fig. S1.** mRNA levels of TFRC, MDM2 and VCAM1. (A, B, C) No significant change in mRNA levels of TFRC, MDM2 and VCAM1 was observed between 275 CRC tumors versus 41 normal colon tissue samples. GEPIA2 was used to analyze TCGA and GTEx datasets. NS: no significance. **Fig. S2.** miR-378a failed to bind to the 3’UTR of c-MYC. (A, B) Luciferase activities of the reporter constructs containing the 3’UTR of c-MYC after treatment with MC-miR-378 or anti-sense oligo (ASO). HCT116 cells transfected with the empty luciferase reporter vector and MC-miR378-MM or scramble served as control. NS: no significance. Data represent mean ± SEM. two-tailed student t test. ns: no significance. **Fig. S3.** MC-miR378 injection into mice led to increased miR-378a in the colon. (A) mRNA levels of FOXQ1 and ODC1 in DLD-1 cells transfected with MC-miR-378a or MC-miR389-MM (two-tailed student t test). (B) Levels of PUT, SPD and SPM in DLD-1 levels treated with MC-miR378 or MC-miR378-MM (two-tailed student t test). (C-D) mRNA levels of FOXQ1 and ODC1 and levels of PUT, SPD and SPM in DLD-1 cells transfected with either MC-miR378-MM (control), MC-miR378, or a combination of MC-miR378 and TPs of ODC1 and FOXQ1. Data represent mean ± SEM. *p < 0.05, **p < 0.01 (two-way ANOVA test). **Fig. S4.** Level of miR-378a in colons of mice treated with MC-miR378. Increased miR-378a in colons of mice treated with MC-miR378 (n=6) compared to the mice treated with MC-miR378-MM (n=6). Data represent mean ± SEM. ***p < 0.001 (two-tailed student t test). **Fig. S5.** Kaplan–Meier survival curve analysis of FOXQ1 for overall survival of CRC patients. Level of FOXQ1 was negatively correlated with overall survival of CRC patients. GEPIA2 was used for survival analysis of CRC patients in the TCGA dataset.

## Data Availability

All data relevant to the study are included in the article or uploaded as supplementary information. Materials are available as reasonably requested.
