# Supplementary material for: MicroRNA-378a-3p prevents initiation and growth of colorectal cancer by fine tuning polyamine synthesis
Source: Cell Biosci. 2022 Dec 1;12:192. doi: 10.1186/s13578-022-00930-3 (PMC9717536; doi:10.1186/s13578-022-00930-3)
Supplement: Supplementary file 1 — Additional file 1: Fig. S1. mRNA levels of TFRC, MDM2 and VCAM1. (A, B, C) No significant change in mRNA levels of TFRC, MDM2 and VCAM1 was observed between 275 CRC tumors versus 41 normal colon tissue samples. GEPIA2 was used to analyze TCGA and GTEx datasets. NS: no significance. Fig. S2. miR-378a failed to bind to the 3’UTR of c-MYC. (A, B) Luciferase activities of the reporter constructs containing the 3’UTR of c-MYC after treatment with MC-miR-378 or anti-sense oligo (ASO). HCT116 cells transfected with the empty luciferase reporter vector and MC-miR378-MM or scramble served as control. NS: no significance. Data represent mean ± SEM. two-tailed student t test. ns: no significance. Fig. S3. MC-miR378 injection into mice led to increased miR-378a in the colon. (A) mRNA levels of FOXQ1 and ODC1 in DLD-1 cells transfected with MC-miR-378a or MC-miR389-MM (two-tailed student t test). (B) Levels of PUT, SPD and SPM in DLD-1 levels treated with MC-miR378 or MC-miR378-MM (two-tailed student t test). (C-D) mRNA levels of FOXQ1 and ODC1 and levels of PUT, SPD and SPM in DLD-1 cells transfected with either MC-miR378-MM (control), MC-miR378, or a combination of MC-miR378 and TPs of ODC1 and FOXQ1. Data represent mean ± SEM. *p < 0.05, **p < 0.01 (two-way ANOVA test). Fig. S4. Level of miR-378a in colons of mice treated with MC-miR378. Increased miR-378a in colons of mice treated with MC-miR378 (n=6) compared to the mice treated with MC-miR378-MM (n=6). Data represent mean ± SEM. ***p < 0.001 (two-tailed student t test). Fig. S5. Kaplan–Meier survival curve analysis of FOXQ1 for overall survival of CRC patients. Level of FOXQ1 was negatively correlated with overall survival of CRC patients. GEPIA2 was used for survival analysis of CRC patients in the TCGA dataset. [file 13578_2022_930_MOESM1_ESM.docx]

**Supplemental material**

Supplemental material including materials and methods, supplementary figures, and figure legends could be found with this article online.

**Gene Expression Analysis in TGCA Database**

GEPIA2 was used for analyzing levels of *ODC1* and *FOXQ1* in 275 CRC tumors and 42 normal colon samples from the TCGA database, using a standard processing pipeline.[^1^](#_ENREF_1)

**Chromatin Immunoprecipitation (ChIP) Assays**

ChIP Assay Kit (Abcam) was used to perform ChIP to precipitate DNA fragments containing FOXQ1 binding site within the promoter of *c-MYC*. Chromatin from DLD-1 cells were used for ChIP. The detailed procedures were performed as described previously.[^2^](#_ENREF_2)

**Identification of miR-378a Targets**

To identify genes with binding motifs for miR-378a, we downloaded the target gene databases of miR-378a based on TargetScan,[^3^](#_ENREF_3) Pictar,[^4^](#_ENREF_4) and Starbase.[^5^](#_ENREF_5) Only hits from TargetScan or PicTar algorithm that were confirmed by Ago HITS-CLIP (high-throughput sequencing of RNAs isolated by crosslinking immunoprecipitation from Argonaute protein complex) were selected. These three databases were compared using Microsoft Access. Our prediction from *in silico* algorithms showed that 3' UTRs of both human and mouse *FoxQ1* and *Odc1* mRNAs are 100% complementary to the miR-378a 5' seed region, exhibiting the highest prediction scores and binding energy.

**Reporter Vector Construction and Luciferase Assay**

To generate the luciferase reporter vectors of 3’UTRs, 3' UTRs of *Odc1*, *c-Myc* and *FoxQ1* were amplified from mouse or human cDNA using PCR, and inserted into the pMiR-Reporter vector (Ambion), referred as pMiR-Odc1, pMiR-FoxQ1, and pMiR-cMYC. Two bases of the binding sites for miR-378a within the 3’UTRs of *Odc1* and *FoxQ1* were mutated using QuikChange II Site-Directed Mutagenesis Kit (Agilent Technologies) per the manufacturer’s instruction, and referred to as pMiR-Odc1-M and pMiR-FoxQ1-M. 24 hours before transfection, 5×10^4^ HCT116 cells were plated per well in a 24-well plate. Then, 200 ng of the luciferase reporter vector and MC-miR378 (100 ng) as well as 30 ng of β-gal plasmid pSV-β-Galactosidase Vector (Promega) were transfected into HCT16 cells using Lipofectamine 2000 (Invitrogen). MC-miR378-MM was used as the control for MC-miR378. 48 hours post transfection, luciferase and β-galactosidase assays were done using the Luciferase Assay System and Beta-Glo® Assay System (Promega). Luciferase activities were normalized to galactosidase activities; wells were transfected in triplicate; and each well was assayed in triplicate.

To generate the luciferase reporter vector of *c-MYC* promoter, the promoter of *c-MYC* was amplified from human genomic DNA using PCR, and inserted into the pGL3-basic (Promega), and referred to as pGL3-cMYC. Two bases of the binding site for FOXQ1 within the promoter of *c-MYC* were mutated using QuikChange II Site-Directed Mutagenesis Kit (Agilent Technologies) per the manufacturer’s instruction, and referred to as pGL3-cMYC-Mu. 24 hours before transfection, 5×10^4^ HCT116 cells were plated per well in a 24-well plate. Then, pGL3-cMYC (200 ng), pCDNA3.1-FOXQ1 (200 ng) and 30 ng of β*-gal* plasmid pSV-β-Galactosidase were transfected into HCT116 cells using Lipofectamine 2000 (Invitrogen). HCT116 cells treated with pGL3-cMYC and empty vector was used as control. After 24 hours of transfection, luciferase and β-galactosidase assays were done using the Luciferase Assay System and Beta-Glo^®^ Assay System (Promega). Luciferase activities were normalized to galactosidase activities; wells were transfected in triplicate; and each well was assayed in triplicate.

**Histological Analysis**

Colons were examined using 4 μm thick, serial sections stained with hematoxylin and eosin. For immunohistochemistry, colon sections were deparaffinized, hydrated and incubated in 3% hydrogen peroxide, to block endogenous peroxidase. Antigen retrieval was performed by heating in 10 mM sodium citrate buffer (pH 6.0) for 10 min using microwave. Specimens were blocked in Protein Block solution (Dako) for 30 min at room temperature (RT) followed by incubation with primary antibody at 4 ℃ overnight. Anti-ki67 (ab15580) was purchased from Abcam. Lesion scores were assigned for inflammation and hyperplasia. Inflammation was scored based on the degree of leukocyte infiltration and the extent of inflammation in the different layers of the colon. Hyperplasia was scored based on the severity of the cellular changes from mild atypia to severe dysplastic changes.[^6^](#_ENREF_6) The detailed scoring criteria were described in a previous publication.[^6^](#_ENREF_6) The degree of adenocarcinoma represents the percentage of adenocarcinoma in lesions.[^6^](#_ENREF_6)

**Target Protector Treatment of DLD-1 Cells**

To determine whether *ODC1* mediates the inhibitory effect of miR-378a on colony formation, would healing, cell viability, and apoptosis, three groups of DLD-1 cells (0.5 × 10^6^ cells in 35 mm plastic dishes) were transfected with MC-miR378-MM, MC-miR378 or a combination of MC-miR378 and Target Protectors (TPs) of *FOXQ1* and *ODC1* (Exiqon, Woburn, MA) (20 nM). Wound healing, cell viability, colony formation and apoptosis were carried out as described in the manuscript.

**RNA Isolation and Quantitative Reverse Transcription-PCR (qRT-PCR)**

Total RNA was isolated with miRNeasy Mini Kit (Qiagen). To assess gene expression, 1 μg RNA was used for cDNA synthesis with Superscript III reverse transcription reagent (Invitrogen). PCR amplification was performed at 50°C for 2 minutes and 95°C for 10 minutes, followed by 40 cycles at 95°C for 15 seconds and 60°C for 1 minute in a 7900 real time-PCR system with SYBR green (Applied Biosystems). For each sample, we analyzed β-actin, GAPDH or 18S rRNA expression to normalize target gene expression. Primers for qRT-PCR were designed with Primer Express software (Applied Biosystems).

To determine levels of miRNA expression, 10 ng RNA were used for miRNA-specific cDNA synthesis with the TaqMan MicroRNA Reverse Transcription Kit and Taqman MicroRNA Assays (all Applied Biosystems). PCR amplification was performed at 95°C for 10 minutes, followed by 40 cycles at 95°C for 15 seconds and 60°C for 1 minute in a 7900 real time-PCR system (Applied Biosystems). The small RNA Sno202 and RNU6 were used to normalize target miRNA expression. Relative changes in gene and miRNA expression were determined using the 2^-ΔΔCt^ method.[^7^](#_ENREF_7)

**Western Blots and Antibodies**

Proteins were extracted in RIPA buffer (Cell Signaling Technology) with proteases inhibitors (Roche). Protein concentration was measured by Pierce BCA Protein Assay Kit and 25~50 μg of total lysate was loaded and immunoblotted for regular Western blot. Anti-FOXQ1 (ab51340), anti-MYC (ab32072) and anti-ODC1 (ab185690) were purchased from Abcam. Anti-actin (NB600-501) was purchased from Novus Biologicals.

**References:**

1. Tang Z, Kang B, Li C, et al. GEPIA2: an enhanced web server for large-scale expression profiling and interactive analysis. Nucleic Acids Research 2019;47:W556-W560.

2. Song G, Wang L. A conserved gene structure and expression regulation of miR-433 and miR-127 in mammals. PloS ONE 2009;4:e7829.

3. Friedman R, Farh K, Burge C, et al. Most mammalian mRNAs are conserved targets of microRNAs. Genome Research 2009;19:92-105.

4. Krek A, Grün D, Poy M, et al. Combinatorial microRNA target predictions. Nature Genetics 2005;37:495-500.

5. Yang J-H, Li J-H, Shao P, et al. starBase: a database for exploring microRNA–mRNA interaction maps from Argonaute CLIP-Seq and Degradome-Seq data. Nucleic Acids Research 2011;39:D202-D209.

6. Jin B-R, Chung K-S, Lee M, et al. High-fat diet propelled AOM/DSS-induced colitis-associated colon cancer alleviated by administration of Aster glehni via STAT3 signaling pathway. Biology 2020;9:24.

7. Schmittgen TD, Livak KJ. Analyzing real-time PCR data by the comparative C(T) method. Nature Protocol 2008;3:1101-8.

**
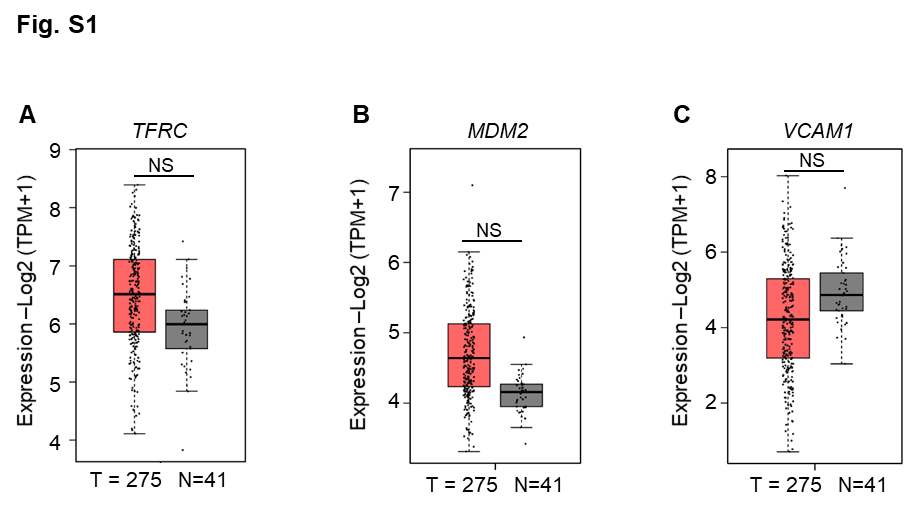
**

**Fig. S1 mRNA levels of *TFRC*, *MDM2* and *VCAM1*. (A, B, C)** No significant change in mRNA levels of *TFRC*, *MDM2* and *VCAM1* was observed between 275 CRC tumors versus 41 normal colon tissue samples. GEPIA2 was used to analyze TCGA and GTEx datasets. NS: no significance.

**
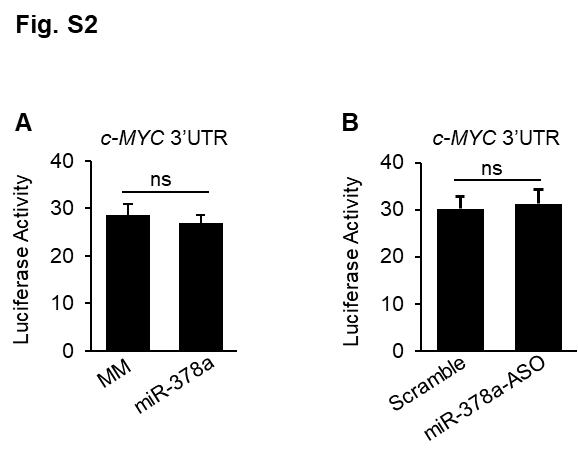
**

**Fig. S2 miR-378a failed to bind to the 3’UTR of *c-MYC*. (A, B)** Luciferase activities of the reporter constructs containing the 3’UTR of *c-MYC* after treatment with MC-miR-378 or anti-sense oligo (ASO). HCT116 cells transfected with the empty luciferase reporter vector and MC-miR378-MM or scramble served as control. NS: no significance. Data represent mean ± SEM. two-tailed student *t* test. ns: no significance


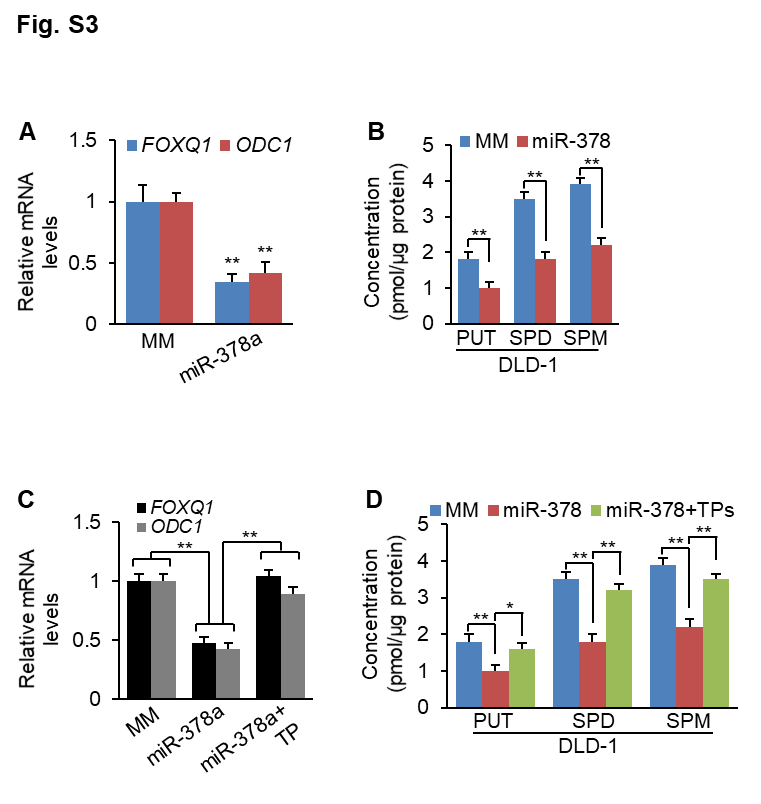


**Fig. S3 MC-miR378 injection into mice led to increased miR-378a in the colon. (**A) mRNA levels of *FOXQ1* and *ODC1* in DLD-1 cells transfected with MC-miR-378a or MC-miR389-MM (two-tailed student *t* test). (**B**) Levels of PUT, SPD and SPM in DLD-1 levels treated with MC-miR378 or MC-miR378-MM (two-tailed student *t* test). (**C-D**) mRNA levels of *FOXQ1* and *ODC1* and levels of PUT, SPD and SPM in DLD-1 cells transfected with either MC-miR378-MM (control), MC-miR378, or a combination of MC-miR378 and TPs of *ODC1* and *FOXQ1*. Data represent mean ± SEM. ^*^*p* < 0.05, ***p* < 0.01 (two-way ANOVA test).

**
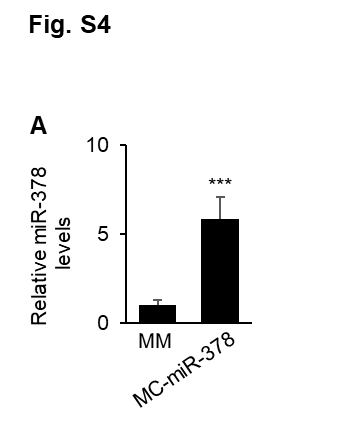
**

**Fig. S4 Level of miR-378a in colons of mice treated with MC-miR378.** Increased miR-378a in colons of mice treated with MC-miR378 (*n*=6) compared to the mice treated with MC-miR378-MM (*n*=6). Data represent mean ± SEM. ****p* < 0.001 (two-tailed student *t* test)

**
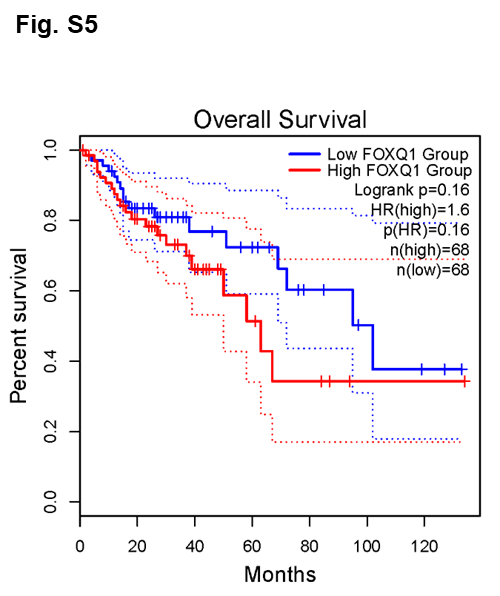
**

**Fig. S5 Kaplan–Meier survival curve analysis of *FOXQ1* for overall survival of CRC patients.** Level of *FOXQ1* was negatively correlated with overall survival of CRC patients. GEPIA2 was used for survival analysis of CRC patients in the TCGA dataset.
